# Recent Insights on the Role of PPAR-β/δ in Neuroinflammation and Neurodegeneration, and Its Potential Target for Therapy

**DOI:** 10.1007/s12017-020-08629-9

**Published:** 2020-11-18

**Authors:** Anna K. Strosznajder, Sylwia Wójtowicz, Mieszko J. Jeżyna, Grace Y. Sun, Joanna B. Strosznajder

**Affiliations:** 1 Faculty of Medicine, Medical University of Bialystok, 1 Kilinskiego st., 15-089 Białystok, Poland; 2 Department of Cellular Signaling, Mossakowski Medical Research Centre Polish Academy of Sciences, 5 Pawińskiego st., 02-106 Warsaw, Poland; 3 Biochemistry Department, University of Missouri, Columbia, MO 65211 USA

**Keywords:** PPAR delta, Agonists, Lipid metabolism, Neurodegenerative disorders, Neuroprotection, Hypoxia/ischemia, Alzheimer's disease

## Abstract

Peroxisome proliferator-activated receptor (PPAR) β/δ belongs to the family of hormone and lipid-activated nuclear receptors, which are involved in metabolism of long-chain fatty acids, cholesterol, and sphingolipids. Similar to PPAR-α and PPAR-γ, PPAR-β/δ also acts as a transcription factor activated by dietary lipids and endogenous ligands, such as long-chain saturated and polyunsaturated fatty acids, and selected lipid metabolic products, such as eicosanoids, leukotrienes, lipoxins, and hydroxyeicosatetraenoic acids. Together with other PPARs, PPAR-β/δ displays transcriptional activity through interaction with retinoid X receptor (RXR). In general, PPARs have been shown to regulate cell differentiation, proliferation, and development and significantly modulate glucose, lipid metabolism, mitochondrial function, and biogenesis. PPAR-β/δ appears to play a special role in inflammatory processes and due to its proangiogenic and anti-/pro-carcinogenic properties, this receptor has been considered as a therapeutic target for treating metabolic syndrome, dyslipidemia, carcinogenesis, and diabetes. Until now, most studies were carried out in the peripheral organs, and despite of its presence in brain cells and in different brain regions, its role in neurodegeneration and neuroinflammation remains poorly understood. This review is intended to describe recent insights on the impact of PPAR-β/δ and its novel agonists on neuroinflammation and neurodegenerative disorders, including Alzheimer’s and Parkinson’s, Huntington’s diseases, multiple sclerosis, stroke, and traumatic injury. An important goal is to obtain new insights to better understand the dietary and pharmacological regulations of PPAR-β/δ and to find promising therapeutic strategies that could mitigate these neurological disorders.

## Introduction

Peroxisome proliferator-activated receptors (PPAR) belong to the family of hormone and lipid-activated nuclear receptors, which are involved in metabolism of cholesterol, sphingolipids, and fatty acids. The transcriptional activity of PPARs is known to engage in a variety of cellular functions including cell differentiation, proliferation, and development (Hong et al. 2019). These receptors heterodimerize with retinoid X receptor (RXR), and the dimer regulates gene expression in response to dietary-derived fatty acids as well as exogenous agonists. Activation of these receptors by endogenous or exogenous ligands can evoke transduction of signals and induce interaction with lipoproteins, coactivators, or corepressors (Evans and Mangelsdorf 2014; Varga et al. 2011). PPARs not only play a role on regulating lipid metabolism and signaling, but also for maintenance of carbohydrates and glucose homeostasis.

Similar to PPAR-α and PPAR-γ in this family, PPAR-β/δ, which is also known as PPAR-δ, was cloned from the mouse genome and identified as an orphan nuclear receptor in the 90 s (Hong et al. 2019). Subsequently, two existing isoforms of this protein were identified by alternative splicing of gene NR1C2. PPAR-β/δ contains the canonical structure domains common to other nuclear receptor family members, including the amino-terminal AF-1 trans-activation domain, a DNA-binding domain, and a dimerization and ligand-binding domain with a ligand-dependent trans-activation function AF-2 at the carboxy-terminal region (Azhar 2010). The amino-terminal AF-1 trans-activation domain is responsible for transcriptional activation. It provides constitutive activation function independent of ligand binding. The DNA-binding domain (DBD, domain C), which is comprised of two zinc-finger motifs, is involved in DNA recognition and protein–protein interaction. While the hinge domain (domain D) is succeeded by the C-terminal Ligand-binding domain (LBD, domains E/F), which contains not only the ligand-binding pocket, but also regions important for dimerization and the AF-2 domain. Ligand binding is thought to induce structural changes in the AF-2 domain, allowing the recruitment of co-activator proteins important for transcriptional activation, thereby serving as a switch to activate PPARs (Brunmeir and Xu 2018). So far, only one post-translational modification for PPAR-β/δ is known. Koo and colleagues showed that PPAR-β/δ SUMOylation at K104 is removed by SUMO-Specific Protease 2 (SENP2) and this promotes the expression of FAO genes in muscle (Koo et al. 2015).

**Fig. 1:**
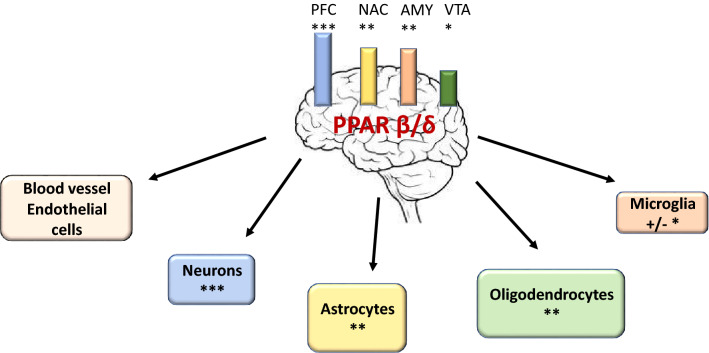
PPAR-β/δ expression in different brain parts and cells. *PFC* prefrontal cortex, *NAC* nucleus accumbens, *AMY* amygdala, *VTA* ventral tegmental area; ***, **, * level of PPAR-β/δ expression; on the basis of data described by Warden et al. (2016), Schnegg and Robbins (2011), Carnigila et al. (2013)

PPAR-β/δ is comprised of 441 amino acids with a molecular weight of 49.9 kDa. According to Gene Cards, this protein is widely expressed and detected in human tissues, including the brain, pancreas, liver, and heart (Hong et al. 2019). Although PPAR-β/δ is expressed in cells in all brain regions, neurons appear to have the highest expression. Warden et al. (2016) demonstrated localization of PPAR isotypes in the adult mouse and human brain (Fig. 1). Using quantitative PCR and double immunofluorescence microscopy, investigation among brain parts indicated highest level of mRNA and proteins in the prefrontal cortex (Warden et al. 2016). In the brain, although all PPAR isoforms have been detected in neuronal and astrocytes, PPAR-β/δ appeared to have low immunoreactivity in microglia as compared with other PPARs members. Analysis of subcellular localization indicated that PPAR-β/δ in neurons is present both in the cytoplasm and nucleus. Nevertheless, its intracellular localization may change depending on patho-physiological conditions and applied therapy (Gamdzyk et al. 2018).

Until recently, studies on the role of PPAR-β/δ were largely carried out with peripheral organs/tissue (Phua et al. 2020). Its expression is detected at early stage of embryogenesis, and disruption of this gene is lethal due to severe placental defects. The knockout animals are characterized by alterations of skin and fat mass, and impairment of brain development. PPAR-β/δ seems to play a key role in embryo development, and its deletion can induce a high rate of mortality around embryonic day 10.5 (E10.5) (Hall et al. 2008; Nadra et al. 2006). At this time of development, the expression of PPAR-β/δ could be detected in all brain regions, including the cerebral cortex, thalamus, cerebellum and brainstem, and reaching peak levels between E 13.5 and E 15.5 (Gofflot et al. 2007; Braissant and Wahli 1998). The expression of PPAR-β/δ was found in neurons, astrocytes, oligodendrocytes, and recently, also in microglia cells (Schnegg and Robbins 2011; Carniglia et al. 2013). In addition, this receptor is also expressed in brain capillary endothelial cells, suggesting an involvement in regulation of blood/brain barrier (Akanuma et al. 2008). Studies using genetically modified PPAR-β/δ null mice indicated changes in brain weight, and concomitantly, the body weight was also smaller as compared to the wide-type control (Peters et al. 2000). Histological study showed disturbances in myelination in the corpus callosum, more frequently in females comparing to males (Markham et al. 2009).

## Role of PPAR-δ in Lipid Metabolism and Signaling Pathways

**Fig. 2:**
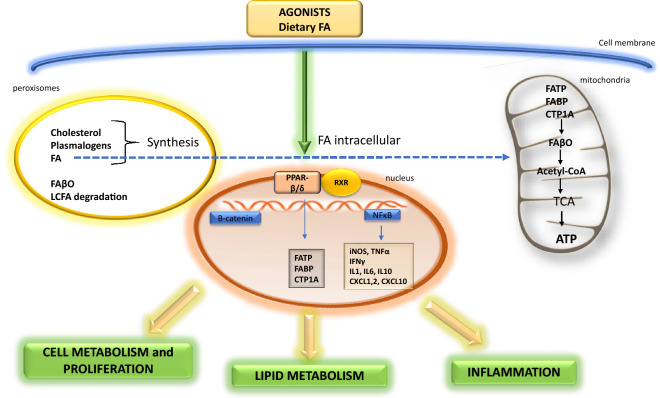
The role of PPAR-β/δ in lipid metabolism. *FA* fatty acids, *FAβO* fatty acids-β oxidation, *FATP* fatty acids transport protein, *FABP* fatty acids binding protein, *CPT1A* carnitine palmitoyltransferase I, *RXR* retinoid X receptor, *ATP* adenosine triphosphate, *TCA* tricarboxylic acid, *acetyl-CoA* acetyl coenzyme A, *LCFA* long-chain fatty acids, *CXCL1,2,10* chemokines 1,2,10, *iNOS* nitric oxide synthases (inducible form), *TNF-α* tumor necrosis factor α, *IFNγ* interferon gamma, *IL1,6,10* interleukin 1,2,10, *NFκB* nuclear factor kappa-light-chain-enhancer of activated B cells

During the past decade, most studies on PPAR-β/δ were carried out with muscle and other peripheral tissue/organs, and relatively few studies were carried out with the brain (Grimaldi 2007; Phua et al. 2020; Wang et al. 2020). The study by Rosenberger et al. (2002) showed significant alterations of phospholipids and esterified fatty acids, together with gender differences in the brain of PPAR-β/δ null mice. Results with PPAR-β/δ null mice also showed defects in brain peroxisomal acyl-CoA utilization and thus projected a role in myelination. PPAR-β/δ also can influence genes engaged in enzymes responsible for fatty acid β oxidation pathway in mitochondria and peroxisome (Grimaldi 2007; Lamichane et al. 2018). Information in Fig. 2 demonstrates the potential roles of PPAR-β/δ in lipid metabolism in the brain. In many instances, these roles are comparable to those demonstrated in hepatocytes and in some tumor cells (Beyaz and Yilmaz 2016).

PPAR-β/δ can alter brain membrane phospholipids, through post-translational modification via the acylation process. This process may lead to changes in protein functions, such as the myelin proteolipid proteins (PLP) (Campagnoni and Macklin 1988). On the other hand, activation of PPAR-β/δ by long-chain saturated and unsaturated fatty acids (LCSFA, LCUFA) and their metabolites may lead to regulation/modulation of transcription of genes encoding proteins such as fatty acid binding proteins (FABP) and fatty acid translocase (FAT).

PPAR-β/δ is also engaged in regulation of cholesterol release and metabolism. However, despite that adult CNS contains 23% of the total sterol pool in the entire body, little information is available regarding this receptor and cholesterol in the brain (Dietschy and Turtley 2004). Cholesterol is an important constituent of the plasma membrane and is the major component of myelin in adult human brain where it consists 70–80% of the whole brain cholesterol. In human adult brain, cholesterol level reaches 23 mg/g w.bw, however, at birth only 6 mg/g bw and in adult mouse brain about 18 mg/g bw. With participation of apolipoprotein E and A, PPAR-β/δ may alter cholesterol metabolism in the brain, and exert effects on neural and glial cells differentiation. However, the relationship between cholesterol metabolism, PPARs, and neurodegeneration/neuroinflammation is till now not fully elucidated.

In the peripheral system, PPAR-β/δ agonists have been proposed for treatment of metabolic syndrome (MetSD) which is tightly connected with long-chain fatty acid (LCFA) homeostasis (Varga et al. 2011). Nevertheless, molecular sequence of events leading to imbalance of lipid homeostasis is still not well understood. It is suggested that higher levels of circulating LCFA and their availability can induce fat accumulation in adipose tissue, liver, and other tissues, leading to insulin resistance and DMT2. Activation of PPAR-β/δ in animal model leads to improvement of lipid homeostasis and insulin sensitivity (Tanaka et al. 2003). There is evidence that LCFA signaling is mediated by PPAR- β/δ, which also plays a crucial role in lipid absorption and intestinal physiology. Due to the proangiogenic and pro-/anti-carcinogenic properties of PPAR-β/δ ligands, these compounds may serve as therapeutic agents for treating metabolic syndrome, dyslipidemia, and diabetes (Bishop-Bailey and Swales 2008).

PPAR-β/δ plays a crucial role in diseases associated with alterations of lipid and glucose metabolism, including MetSD, DMT2, and atherosclerosis. MetSD is a complex pathological condition together with dyslipidemia, hyperglycemia, central obesity, and hypertension, many are associated with prothrombotic and proinflammatory state. The study by Serrano-Marco et al. (2011) suggested that PPAR-β/δ activation could impede IL6-induced STAT3 activation by inhibition of ERK1/2 and prevention of STAT3 association with Hsp90. This effect may contribute to the suppression of cytokine-induced insulin resistance in adipocytes and possibly may also occur in the brain. Obviously, more studies are needed to better understand the role of this receptor in neurodegenerative and neuroinflammatory diseases.

## Role of PPAR-β/δ in Oxidative Stress and Neuroinflammation

PPARs are known to modulate inflammatory processes associated with lipid signaling pathways. Suppressing inflammatory processes in the CNS could lead to reduction of brain damage and improvement of motor and cognitive outcome (Villapol 2018). Resident microglia and infiltrated inflammatory cells were regarded as mediators responsible for this process (Salvi et al. 2017).

PPAR-β/δ is not only a lipid sensor, but also a regulator of mitochondrial function, and may influence oxidative stress and inflammation in brain cells as well as proliferation and angiogenesis in vascular endothelial cells (Bishop-Bailey and Swales 2008). There is evidence that PPAR-β/δ regulates vascular function by enhancing VEGFR expression, phosphorylation of AKT, and subsequently regulating endothelial NO production and reducing ROS and inflammation (Jiang et al. 2019).

Systemic inflammatory responses (SIR) evoked by the endotoxin lipopolysaccharide (LPS) may contribute to neurodegenerative disorders (Brown 2019). PPARs are unique set of fatty acid regulated transcription factors controlling both inflammation and lipid metabolism (Varga et al. 2011; Schnegg and Robbins 2011). It was recently reported that PPAR-β/δ agonists exerted significant anti-inflammatory effects and suppressed the genes encoding iNOS, several chemokines such as CXCL1, CXCL2, CXCL10 interleukins IL1, IL6, and other cytokines TNF-α, IFN-γ, and concomitantly enhanced IL10 (Kuang et al. 2012; Chehaibi et al. 2017; Beyaz and Yilmaz 2016). Agonist of PPAR β/δ such as GWO 742 could decrease neutrophil infiltration into the brain during ischemia and protects against neuroinflammation (Chehaibi et al 2017). However, activation of other members of PPARs evoked also anti-inflammatory effect (Varga et al. 2011; Carniglia et al. 2013; Villapol 2018).

In our previous studies, acute SIR evoked by LPS administered i.p. to mice-induced memory impairment showed alterations of transcription of pro-oxidative, inflammatory genes, and genes engaged in cells death signaling (Czapski et al. 2010; 2016; Jacewicz et al. 2009). During the last decade, there has been increasing interest on the involvement of PPAR-β/δ in inflammatory processes (Bishop-Bailey and Bystrom 2009; Piqueraset al 2009; Schnegg and Robbins 2011).

## PPAR-β/δ in AD and Other Neurodegenerative Disorders

### Alzheimer’s Disease (AD)

Alzheimer’s disease (AD) is the most prevalent, progressive, and irreversible neurodegenerative disease that leads to dementia. There are many underlying mechanisms towards the pathogenesis of AD, including the widely known amyloid pathogenesis with liberation and oligomerization of amyloid beta peptides (Aβ), and hyperphosphorylation of the microtubule-associated tau protein, and its polymerization into insoluble, neuronal fibrillary tangles (NFTs). These alterations lead to astrocytes and microglia cells activation, and consequently, inflammatory response (Selkoe and Hardy 2016). On the cellular level, alterations of mitochondrial activity/ function and increase of oxidative stress may play a crucial role in AD pathogenesis (Tiwari et al. 2019; Schmitt et al. 2012; Swerdlow 2018). Recent studies further indicated and suggested that abnormal sphingolipids, phospholipids, and fatty acids metabolism could be early and key events in the pathogenesis of AD (Kunkle et al. 2019; Cuyvers and Sleegers 2016; Jęśko et al. 2019a, b; Picard et al. 2018). Among these lipids, fatty acids and their metabolites through specific receptors and PPARs signaling are engaged in regulation of brain function, learning and memory. Recent studies have described the essential role of PPAR-α in regulation of lipid metabolism, neuronal function, synaptic plasticity, and cognition (Wójtowicz et al. 2020; Sáez-Orellana et al. 2020). Due to the complexity of AD pathophysiology, there is advantage for testing agonists that target different isoforms of PPARs (Reich et al. 2019).

**Fig. 3:**
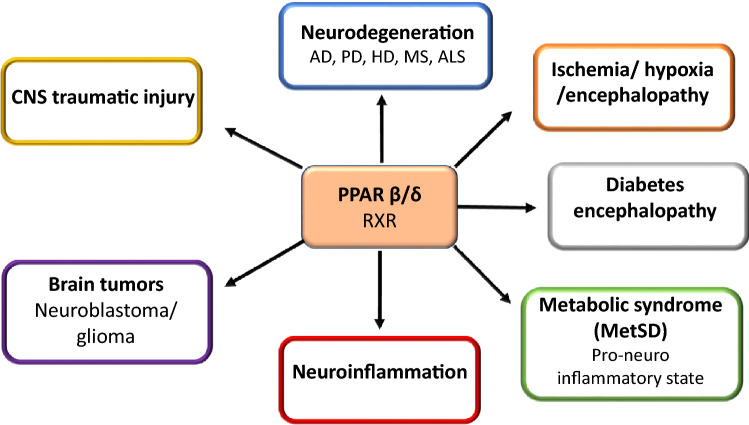
PPAR-β/δ engagement in neurological disorders. *AD*-Alzheimer’s disease, *PD* Parkinson’s disease, *HD* Huntington’s disease, *MS* multiplex sclerosis, *ALS* amyotrophic lateral sclerosis

Previous studies have shown that downregulation of PPAR-β/δ could be linked to both neuroinflammation and insulin resistance in the brain (de la Monte and Wands 2006). Alzheimer’s disease is often regarded as a brain form of diabetes, and insulin deficiency or resistance to insulin may lead to neurodegeneration (Tong et al. 2016a, b). Insulin plays a fundamental role in regulating Extracellular Signal-Regulated Kinases (ERK), which are essential for learning and memory, and are compromised in early AD (Dineley et al. 2014). Therefore, maintaining the action of insulin in the brain could potentially restore brain function and reduce neurodegeneration (Tong et al. 2016a, b; Jęśko et al. 2019a, b). PPARs are known to modulate insulin-stimulated gene expression, by responding to signals that are transmitted from surface cells membranes (Collino et al. 2008). As compared to other PPARs, PPAR-β/δ seems to be most expressed in the brain (Cimini et al. 2005), and expression of PPAR-β/δ was reduced in the brains of AD patients similar as PPAR-α but the expression of gene for PPAR-γ was selectively upregulated (de la Monte and Wands 2006) (Fig. 3).

A new PPAR-δ/γ agonist (T3D-959) with 15-fold higher PPAR-β/δ selectivity/potency (comparing to PPAR-γ) is in an exploratory phase II clinical trial on thirty-four mild-to-moderate AD patients. (Chamberlain et al. 2020). Due to PPAR-β/δ, PPAR-γ activation this agonist might have synergistic/ additive effects on glucose metabolism and regulation of glucose homeostasis in the brain (Chamberlain et al. 2020). In a previous study, T3D-959 administration was shown to significantly improve motor functions and normalize structure of white matter in streptozotocin (STZ)-induced animal model of sporadic AD (intra-cerebrally injected STZ). The data also showed good blood–brain barrier penetration, good therapeutic index, and high brain concentration for this compound (Tong et al. 2016a, b). This compound also effectively restored integrity of temporal lobe, hippocampal structure, and IGF-1 sensitivity and inhibited neuroinflammation (de la Monte et al 2017; Malm et al. 2015; Tong et al. 2016a, b). Results from the latest phase of the study showed that T3D-959 is generally safe and well tolerated by AD patients (Tong et al. 2016a, b). Plasma metabolome profile indicated dose-related systemic effects on insulin-related metabolism. Moreover, relative FDG-PET imaging displayed regional, dose-dependent effects of this compound on cerebral metabolic rate of glucose. Studies on cognitive assessments (ADAS-cog11 and DSST) indicated improvements with possible pharmacodynamics related to T3D-959 mechanism of action. Due to the encouraging results of the phase II clinical trial, this drug warrants further investigation in a larger clinical study with a proper placebo-controlled group (Chamberlain et al. 2020).

**Fig. 4:**
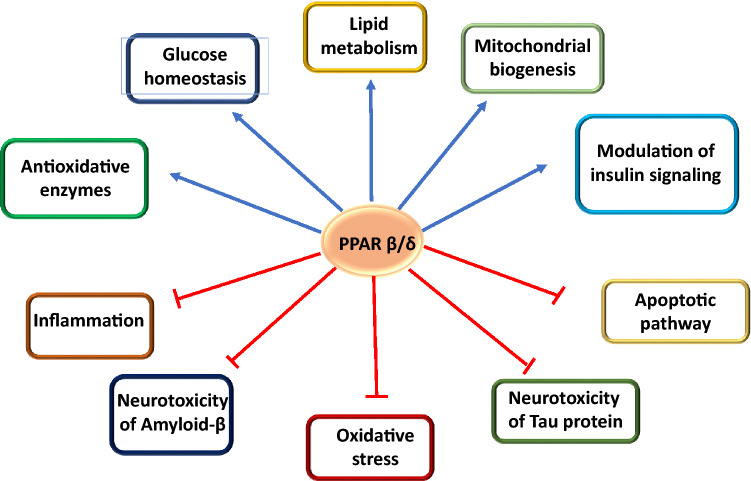
Potential PPAR-β/δ mechanisms of action, constructive in Alzheimer’s disease

The insulin sensitizing action of PPAR-β/δ is probably not the only event with possible impact on AD. As mentioned before, PPAR-β/δ has a potent anti-inflammatory effect and it can stabilize myelin sheath, decline Aβ deposits, as well as exert other molecular effects (Collino et al. 2008; Dunn et al. 2010; Sergey et al. 2009) (Fig. 4). Moreover, experimental depletion of PPAR-β/δ indicated not only increases in neuroinflammation, but also oxidative stress, astrogliosis, and Aβ42 deposition (Barroso et al. 2013).

In a transgenic model of AD (5XFAD mice), the PPAR-β/δ agonist GW0742 could decrease parenchymal Aβ deposits, although intraneuronal Aβ was not affected (Malm et al. 2015). The results of this study showed that this agonist not only significantly decreased Aβ load in the cerebral cortex and hippocampus, but also decreased the level of several cytokines (IL1, IL6, CCL2, and TNF-α) and microglial activity surrounding Aβ deposits. The action of GW0742 was also analyzed in hippocampus of mice with Aβ1-42-induced neurotoxicity (An et al. 2016). Administration of aggregated oligomer of Aβ1-42 (410 pmol/mouse) greatly disrupted memory and learning (in Morris Water Maze and Y-maze tests). This perturbation was associated with decreased expression of PPAR-β/δ in the mouse hippocampus (An et al. 2016). Intra-hippocampal infusion of GW0742 could also reverse the decreased expression of hippocampal PPAR-β/δ, repressed neuroinflammation and apoptotic responses triggered by Aβ1-42 oligomers, and enhanced Bcl2/Bax ratio in hippocampus (An et al. 2016).

PPAR-β/δ and other members of these receptor family are involved in neuroinflammation processes in AD as well as other neurodegenerative disorders. The study of Sergey et al. (2009) showed that the PPAR-β/δ agonist, GW0742 could significantly reduce astrocyte activation, thus exerting anti-inflammatory effect on glial cells. These authors also reported that PPAR-β/δ agonist could reduce amyloid burden, an event presumably mediated by its effect on amyloid clearance.

### Parkinson’s Disease

There is evidence for protective properties of PPAR-β/δ agonists in Parkinson disease (PD) (Chaturwedi and Beal 2008). The study by Iwashita et al. (2007) demonstrated that PPAR-β/δ agonists GW501516 and L165041 exhibited protective function against striatal dopamine depletion induced by 1-methyl-4-phenyl-1,2,3,6-tetrahydropyridine (MPTP). These agonists also inhibited caspase-3 activation, thereby protecting (SHSY-5Y) neuronal cells from action of MPP + (1-methyl-4-phenylpyridinium) and exerted protective effects also in in vivo model of PD. Moreover, the study of Das et al. (2014) demonstrated the effect of PPAR-β/δ agonist—GW0742—in a rat model of PD-associated cognitive impairment. In this study, rats given MPTP resulted in DNA fragmentation and oxidative stress. Subsequent treatment with GW0742 was shown to partially restore cognitive functions impaired by MPTP. Immunochemical (Tunel) assay, and assays of glutathione (GSH) and malondialdehyde (MDA) revealed that GW0742 reduced oxidative stress and DNA fragmentation. In a recent study, intracerebroventricular administration of GW501516, a highly selective agonist for PPAR-β/δ was shown to exert protective effects in PD model induced in mice by MPTP (Chen et al. 2019). In this study, GW501516 not only reduced the movement impairment in the PD mice, it also suppressed dopaminergic neurodegeneration and inhibited activation of the nucleotide-binding domain and leucine-rich-repeat-protein 3 (NLRP3) inflammasome in the astrocytes but not microglia.

### Huntington’s Disease

The study of Dickey et al. (2016) documented that PPAR-β/δ-mediated transcriptional alteration could involve mitochondrial abnormalities and bioenergetic defects in Huntington Disease (HD). The study showed that PPAR-β/δ dysregulation is crucial in the pathogenic cascade of HD and it could elicit neuroprotection in neurons from mouse models of HD. Moreover, treatment with its selective agonist evoked a robust positive response. Through testing PPAR (α, γ and β/δ) individually, and with the use of agonist treatment or shRNA knockdown, this study confirmed the important role of PPAR-β/δ in HD. Experiments on transgenic PPAR-β/δ mice revealed the necessity of PPAR-β/δ for neuronal function (Dickey et al. 2016).

### Multiple Sclerosis

The role of PPAR-β/δ has been studied in Multiple Sclerosis (MS), a disease involving demyelination of central nervous system and affecting nearly one million people worldwide (Dean et al. 1994; Lucchinetti et al. 2011). MS is known for deficiencies in sensory and motor areas, resulting probably from autoimmune mechanism. MS patients also showed changes in plasma lipid profiles, implicating the role of lipids in MS pathogenesis (Weinstock-Guttman et al. 2011). The most widely used model for studies on MS is Experimental autoimmune encephalomyelitis (EAE) (Constantinescu et al. 2011). Studies showed that PPAR-β/δ agonists, through a negative feedback loop, could reduce inflammation and damage of tissues in EAE models of MS (Polak et al. 2005). The study by Drohomyrecky et al. (2019) also demonstrated that mutant mice hypomorphic for PPAR-β/δ receptor showed a more severe course of inflammatory process in CNS, and this event could be revered by PPAR-β/δ agonists (Drohomyrecky et al. 2019).

## PPAR-β/δ in CNS Hypoxia/Ischemia

Neurodegeneration and neuroinflammatory processes play a significant role in brain ischemia/hypoxia pathology. Alteration of lipid metabolism, including polyunsaturated fatty acids and synthesis of several eicosanoids and docosahexanoids were recognized as the early and most important events in ischemia/hypoxia encephalophathy (Bazan 1970; Tang and Sun 1985; Strosznajder and Domanska-Janik 1980; Nalivaeva and Rybnikova 2019). Omega 3 fatty acids supplementation was shown to exert protection via anti-inflammatory action by suppressing microglia response in neonatal hypoxic-ischemic brain injury (Zhang et al. 2010). Study by Saganuma et al. (2013) also demonstrated that docosahexaenoic acid (DHA) supplementation may be beneficial in ischemia hypoxia encephalopathy. In a rodent model of brain ischemia, the data by Song et al. (2019) showed that oleic acid (OA) could mediate neuroprotection through PPAR-γ activation and its anti-inflammatory effect.

Using GW0742, a specific agonist for PPAR-β/δ receptor, the study by Gamdzyk et al. (2018) demonstrated that stimulation of PPAR-β/δ could exert neuroprotective effects in a rat model of neonatal hypoxic–ischemia (HI). In this study, administration of GW0742 reduced brain infarct area, brain atrophy, apoptosis, and improved neurological function at 72 h and 4 weeks post HI. Additionally, GW0742 administration induced several molecular processes, e.g., enhancing the transcription of gene coding PPAR-β/δ, increase in miR-17-5p level, and downregulation of the Thioredoxin Interacting Protein (TXNIP) in the ipsilateral hemisphere. These events also led to inhibition of the Apoptosis Signal-regulating Kinase 1 (ASK1/p38) signaling pathway and reduced apoptotic cell death. In contrary, GSK3787, an antagonist of this receptor, was shown to reverse the protective effects evoked by intranasal administration of GW0742. The study of Hack et al. (2012) and Zaveri et al (2009) demonstrated the effect of PPAR-β/δ antagonists GSK3787, GSK0660, and SR13904, respectively.

In the recent review article, Gamdzyk et al. (2020) compared neuroprotective efficacy of PPAR-β/ δ agonists to PPAR-α and PPAR-γ and conclude that despite of being the most highly expressed in CNS, the available data on the effect of this receptor agonists in stroke as well as other neurological disorders are relatively poor and thus needing further investigations. However, the last results of Chehaibi et al. (2017) demonstrated several ameliorating effects of PPAR-β/δ agonist GW0742 in mice brain ischemia evoked by occlusion of middle cerebral artery (MCA). The significant anti-inflammatory effect was exerted by this agonist, which decreased the neutrophil infiltration, the level of several chemokines and interleukins such as IL-1β, IL6, and other cytokines. Using the same model of brain ischemia. Pialat et al. (2007) previously demonstrated in magnetic resonance imaging (MRI) scan that PPAR-β/δ-null mice comparing to control wild mice indicated significant differences in lesion volume. The effect of GW0742 was also investigated by Tang et al. (2020) in a collagenase-induced intracerebral hemorrhage (ICH) mouse model. In this study, PPAR-β/δ agonist was administered (intraperitoneally in a dose of 3 mg/kg body weight) 30 min before ICH, and its neuroprotective effects included mitigation of behavioral dysfunction and molecular pathways associated with activation of inflammation and apoptosis. A previous study by Paterniti et al. (2010) evaluated the involvement of PPAR-β/δ in spinal cord injury (SCI) in mice evoked by application of vascular clips (force of 24 g) to the dura via a four-level T5 to T8 laminectomy. GW0742 (administered i.p. in a dose of 3 mg/kg body weight) exerted significant neuroprotective effect, and ameliorated the recovery of limb function. The protective effects of this agonist include inhibition of neutrophil infiltration, expression of proinflammatory cytokines and altered molecular processes leading to cell death through changes of transcription of pro- and anti-apoptotic proteins (Fasl, Bax, Bcl2). The protective processes evoked by GW0742 could be eliminated by specific receptor antagonist (GSK0660), which was administered (1 mg/kg bw) at 30 min before GW0742. This high-affinity PPAR-β/δ agonist GW0742 was able to evoke significant neuroprotective effects in secondary damage, during experimental spinal cord injury (SCI) in mice (Paterniti et al. 2010). In this study, GW0742 treatment (0.3 mg/kg^−1^ i.p) at 1 and 6 h after SCI, significantly reduced inflammation, nitric oxide synthesis, nitrotyrosine formation and activation of apoptotic signaling. Moreover, this agonist protected against edema and showed positive effect on motor recovery score. The study of Esposito et al. (2012) indicated that GW0742, through targeting divergent downstream pathways regulating PPAR-β/δ receptors, could decrease changes on both molecular and cellular levels that take place in spinal cord damage. CNS hypoxia–ischemia hemorrhage and traumatic injury are closely connected with vascular alterations, oxidative stress and hypertension. In a preclinical study, Toral et al. (2016) showed antihypertensive effects of PPAR-β/δ in spontaneously hypertensive rats (SHR) as well as in other animal models. Pharmacological activation of PPAR-β/δ exerted several protective effects, improved the endothelial dysfunction, decreased vascular inflammation and vasoconstriction responses. There is evidence that other isoforms of PPARs can also show protective effects on cerebral ischemia damage. In a study by Wu et al. (2016), cultured neurons were subjected to in vitro oxygen–glucose deprivation (OGD), and treatment with GW9662, an antagonist for PPAR-γ, could ameliorate neuronal apoptosis and inhibit p22phox subunit of NADPH oxidase (Wu et al. 2016). It is possible to suggest that agonist acting simultaneously on PPAR-γ and PPAR-β/δ could be more effective in OGD model and in brain ischemia pathology comparing to PPAR-γ alone. Despite of evidence indicating ability for PPAR-β/δ agonists to exert neuroprotective effects on cerebral ischemia injury, there were also negative results, probably depending on the type of agonists used and method of administration. For example, in a study by Knauss et al. (2018) oral administration with SAR 145, a known lipophilic agonist for PPAR-β/δ, could not improve short or long outcomes after focal cerebral ischemia induced to mice through middle cerebral artery occlusion (Knauss et al. 2018).

## PPAR-β/δ in Brain Tumors (Neuroblastomas and Gliomas)

Despite of the recognition of PPAR-β/δ in metabolic and inflammatory diseases, there is increasing interest in developing appropriate ligands/antagonists towards treatment of cancer (Wagner and Wagner 2020; Reil and Lee 2008; Liu et al. 2018). However, the molecular mechanism of PPARs in carcinogenesis is still not fully elucidated, and data from in vitro and in vivo studies are still controversial. Tatenhorst et al. (2008) concluded in their review articles that the agonists of PPARs could be promising for new approaches in human CNS tumor therapy. Subsequently, Youssef and Badr (2011) tried to explain the complexity of these receptor responses and their conformational changes that influence their ability to recruit specific functionally distinct coactivators. For better understanding of the complicated role of PPAR-β/δ in carcinogenesis, these authors recalled the work of Mukherjee et al. (1994), showing that some receptors (such as androgen receptors) exhibited capability to interact with 150 proteins/polypeptides, and thus suggested such a possibility for PPAR in carcinogenesis. It also seems that, in the case of PPAR-β/δ, the complex coactivators and repressors in PPAR-β/δ could be subjected to deeper analysis. Recent studies of Yao et al. (2017) showed that PPAR-β/δ could inhibit human neuroblastoma cell tumorigenesis by inducing protein p-53 and SOX2 mediated cell differentiation. These results suggest that combinatorial activation of retinoic acid receptor, PRAR-α and PPAR-β/δ may be promising therapeutic approach for RA-resistant neuroblastoma patients. Ding et al. (2020) demonstrated the impact of PPAR-β/δ and PPAR-γ polymorphism on glioma risk and prognosis in the Chinese Han population.

## Summary and Perspective

Considering their anti-inflammatory, neuroprotective, and anti-tumors properties, PPAR-β/δ agonists are promising treatments of AD and other neurodegenerative disorders. A list of the natural and synthetic agonists for PPAR-β/δ is shown in Table 1. These PPAR-β/δ ligands should be applied in various other pathologies as DMT2, MetSD, atherosclerosis, obesity, hepatosteatosis. The role of PPAR-β/δ in cancer should be better elucidated and understood. Besides PPAR-β/δ, agonists of PPAR-α and PPAR-γ may also be involve in neurodegenerative diseases, in MetSD and dyslipidemia. Therefore, future studies should test PPAR-β/δ ligands in combination with ligands of other PPARs receptor in these neurological disorders and in inflammation.

**Table 1:** Natural and synthetic agonists of PPAR-β/δ

Natural	Synthetic
	Specific PPAR-β/δ agonists
*PPAR-β/δ agonists*	
Saturated fatty acids – Stearic acids (SA) 18:0 oktadecanoic acid (Korbecki et al. 2019) – Palmitic acid (PA) 16:0 hexadecanoic acid (Korbecki et al. 2019) Monounsaturated fatty acids – Palmitoleic acid 16:1 (*n*-7) cis-hexadec-9-enoic acid (Korbecki et al. 2019) – Oleic acid (OA) 18:1 (*n*-9) cis-octadec-9-enoic acid (Korbecki et al. 2019) Polyunsaturated fatty acids – Docosahexaenoic acid (DHA) 22:6 (*n*-3) all-cis-4,7,10,13,16,19-docosahexaenoic acid (Korbecki et al. 2019) – Eicosapentaenoic acid (EPA) 20:5 (*n*-3) – all-cis-5,8,11,14,17 eicosapentaenoic acid (Korbecki et al. 2019) – Linoleic acid (LA) 18:2 (*n*-6) all-cis—9,12-octadecadienoic acid (Han et al. 2017a, b; Korbecki et al. 2019) – Γ-linoleic acid (GLA) 18:3 (*n*-6) all-cis-6,9,12-octadecatrienoic acid (Korbecki et al. 2019), – Dihomo-γ-linoleic acid (DGLA) 20:3 (*n*-6) 8,11,14-Eicosatrienoic acid gamma- Homolinolenic acid (Han et al. 2017a, b) – Arachidonic acid (AA) 20:4 (*n*-6) all-cis-5,8,11,14 eicosatetraenoic acid (Korbecki et al. 2019) *Arachidonic acid metabolites* – 8(*S*)-HETE 8S-hydroxy-5Z,9E,11Z,14Z-eicosatetraenoic acid (Korbecki et al. 2019) – 15-HETE 15-hydroxyeicosatetraenoic acid (Korbecki et al. 2019) Eicosanoids 15d-PGI2 15-deoxy-∆-^12,14^-prostaglandin J2 PGJ2—prostaglandin J2 PGI2 (prostacyclin)—prostaglandin I2 PGA1/2—prostaglandin A1/A2 PGB2—prostaglandin B2 (Han et al. 2017a, b part I/II)	– L165041 (4-[3{4-Acetyl-3-hydroxy-2-propylphenoxy} propoxyl}] phenoxy)acetic acid; (Han et al. 2017a, b part I/II) – GW501516 2-Methyl-4(((4-methyl-2-(4-trifluoromethyl-phenyl)1,3-thiazol-5-yl) methyl) sulfanyl)phenoxy)acetic acid (Han et al. 2017a, b part I/II) – GW0742 [4-[[[2-[3-Fluoro-4-(trifluoromethyl) phenyl]-4-methyl-5-thiazolyl]thio]-2-methyl phenoxy]acetic acid; (Han et al. 2017a, b part I/II) – GW1929 (2*S*)-((2-Benzoylphenyl)amino-3[4-[2-(methylpyridin-2-ylamino) ethoxy]phenyl)-propionic acid; (Han et al. 2017a, b part I/II) – CER-002 (Han et al. 2017a, b part I/II) – HPP593 (Han et al. 2017a, b Part II) – GW2433 2-[4-(3-{[2-(2-chloro-6-fluorophenyl) ethyl] [(2,3-dichlorophenyl) carbamoyl] amino} propyl) phenoxy]-2-methylpropanoic acid (Li et al. 2018) – MBX-8025 2-[4-[[2R)-2-ethoxy-3-[4 (trifluoromethyl) phenoxy]propyl]thio]-2-methylphenoxy]acetic acid (Han et al. 2017a, b part I/II; Xu et al. 2018; Hong et al. 2019) – Carbaprostacyclin (cPGI) 6,9α-methylene-11α,15S-dihydroxy-prosta-5E,13E-dien-1-oic acid (Han et al. 2017a, b part I/II) – ETYA 5,8,11,14- eicosatetraynoic acid (Korbecki et al. 2019)
	*Dual PPAR-α/βδ agonists* – GFT505 2-(2,6-dimethyl-4-(3-(4-(methylthio) phenyl)-3-oxo-1-propenyl)phenyl)-2-methylpropanoic acid (Han et al. 2017a, b part I/II; Li et al. 2018) – KD-3010 (Xu et al. 2018)
	*Dual PPAR-βδ/γ agonists* – T3D-959 sodium 2-(5-(2-(5-ethyl-2-(4-methoxyphenyl)oxazol-4-yl)ethoxy)-2,3-dihydro-1H-inden-1-yl)acetic acid (Chamberlain et al. 2020) *Pan PPAR-α/βδ/γ agonists* – Chiglitazar l-tyrosine, *O*-[2-(9H-carbazol-9-yl) ethyl]-*N*-[2-(4-fluorobenzoyl)phenyl] (Han et al. 2017a, b part I) – Netoglitazar (Han et al. 2017a, b part I) – Sodeglitazar (Han et al. 2017a, b part I) – Indeglitazar 3-[1-[(4-methoxyphenyl) sulfonyl]-5-methoxy-1H-indole-3-yl] propanoic acid (Han et al. 2017a, b part I) – Sipoglitazar 3-[3-ethoxy-1-[4-(2-phenyl-4-thiazolylmethoxy)benzyl]-1H-pyrazol-4-yl]propionic acid (Han et al. 2017a, b part I) – IVA337 (Li et al. 2018)
*PPAR-β/δ antagonists*	
GSK3787—4-chloro-*N*-[2-[[5-(trifluoromethyl)-2-pyridinyl]sulfonyl]ethyl]benzamide (Paterniti et al. 2010) GSK0660—3-[[[2-methoxy-4-(phenylamino)phenyl]amino]sulfonyl]-2-thiophenecarboxylic acid methyl ester (Paterniti et al. 2010) SR13904 (Zaveri et al. 2009)	
